# Subcutaneous Injection Performance in Yucatan Miniature Pigs with and without Human Hyaluronidase and Auto-injector Tolerability in Humans

**DOI:** 10.1208/s12249-020-01880-0

**Published:** 2021-01-06

**Authors:** Galen H. Shi, Robert J. Connor, David S. Collins, David W. Kang

**Affiliations:** 1 Eli Lilly and Company, Lilly Corporate Center, Indianapolis, Indiana USA; 2 Halozyme Therapeutics, Inc., 11388 Sorrento Valley Road, San Diego, California 92121 USA

**Keywords:** rHuPH20, subcutaneous injection, miniature pigs, auto-injector, pre-filled syringe

## Abstract

Recombinant human hyaluronidase PH20 (rHuPH20) facilitates subcutaneous (SC) delivery of co-administered therapeutic agents by locally and transiently degrading hyaluronan in the SC space, and can be administered with therapeutics using a variety of devices. Two SC delivery studies were carried out to assess auto-injector (AI) performance, each in 18 Yucatan miniature pigs. Abdominal injections were administered using three auto-injectors of 1 mL (AI1) and 2 mL (AI2 and sAI2) with different injection speeds and depths (5.5–7.5 mm) and two pre-filled syringe (PFS) devices of 1 and 2 mL. The injection included a placebo buffer with and without rHuPH20 to evaluate the effect of rHuPH20 on SC injection performance. The feasibility of using similar devices to deliver a placebo buffer in humans was investigated. rHuPH20 was not studied in humans. In miniature pigs, postinjection swelling was evident for most PFS/AI injections, particularly 2 mL. Swelling heights and back leakage were typically lower with rHuPH20 co-administration *versus* placebo for most device configurations (1 or 2 mL PFS or AI). Auto-injections with *versus* without rHuPH20 also resulted in reduced swelling firmness and faster swelling resolution over time. Slow injections with rHuPH20 had shorter and more consistent injection time *versus* placebo. In humans, minimal injection site swelling and negligible back leakage were observed for 2-mL injections of placebo, while more erythema was observed in humans *versus* miniature pigs. Even at high delivery rates with PFS or AI, the addition of rHuPH20 resulted in improved SC injection performance *versus* placebo in miniature pigs.

**Supplementary Information:**

The online version contains supplementary material available at 10.1208/s12249-020-01880-0.

## INTRODUCTION

Parenteral routes for delivering therapeutic agents include intravenous (IV), subcutaneous (SC), intramuscular, and intradermal administration. SC delivery has many potential advantages over IV administration, including shorter administration time, the possibility of self-administration, multiple potential injection sites, low risk of systemic infection, low cost, and reduced infusion-related reactions (1–5). For these reasons, SC administration is often the preferred route of delivery for patients, healthcare professionals, and payers (6–8).

Conventional SC administration has been generally limited to volumes of ≤ 2 mL and has been considered unsuitable for agents that require large injection volumes (9,10). Advances in SC delivery technology enable self-injection of 2 mL using a pre-filled syringe (PFS) and auto-injector (AI), potentially increasing patient choice and compliance (11–15).

One of the barriers for SC administration of large volumes is hyaluronan (HA), a gel-like component of the extracellular matrix that resists bulk fluid flow through the SC space and limits large-volume SC drug delivery and dispersion (16–19). Recombinant human hyaluronidase PH20 (rHuPH20) is a recombinant human form of the naturally occurring human hyaluronidase PH20 enzyme. rHuPH20 facilitates SC delivery of co-administered therapeutic agents by locally and transiently degrading HA in the SC space, overcoming volume limitations of conventional SC delivery and dispersion (20–22). In 2005, the United States Food and Drug Administration approved rHuPH20 as an adjuvant to facilitate SC delivery (23), and multiple therapeutic agents have also received approval for co-formulation or sequential co-administration with rHuPH20 (24–32).

The miniature pig is a non-clinical model that is suitable for evaluating SC administration conditions of biotherapeutics due to its anatomical or physiological similarity to human skin (33,34). Although there are some differences, such as a variably thicker SC fat layer and somewhat higher skin pH in miniature pigs than humans (6–7 compared with 5), many other factors are similar and thereby enhance clinical translatability, including general morphology, epidermal thickness, immunological reactivity, cellular composition, permeability, and metabolic properties (33). Like in humans, the texture and thickness of miniature pig skin varies according to the body site (34,35). The skin of the adolescent miniature pig is particularly translatable to human skin in terms of thickness and structure (33), and the abdominal area is suited for SC administration of larger volume and AI devices. Although SC administration behind the ear in miniature pigs is the most translatable to human skin (34), the bone structure beneath the skin can hinder AI devices and limit the volume that can be administered. As in humans, the dermis of the miniature pig is vascularized, although the vasoconstrictor capability in miniature pigs is more developed (35). Due to these similarities, miniature pigs have been used as a model for assessing dermal inflammation, including erythema and edema responses (36). The general composition of SC tissue is similar in miniature pigs and humans, consisting of adipose lobules in a network of fibrous tissue that connects the dermis and deep muscle layer below the skin (37).

In this study, we investigated the effect of rHuPH20 on the tolerability of placebo buffer injection and device performance in miniature pigs, using 1- and 2-mL auto-injector and PFS devices. Three different auto-injectors of 1 and 2 mL, with different injection speeds and injection depths, and two different PFS devices of 1 and 2 mL were evaluated. The goal was to assess the impact of injection volume, rate, and depth, as well as the effect of rHuPH20 on postinjection swelling, injection backpressure, auto-injector delivery time, and erythema. We also investigated tolerability of placebo buffer, which did not include rHuPh20, using selected, comparable devices in humans in a separate study. Although the miniature pig and human studies were not designed to be directly comparable, and the solutions injected differed between the studies, the clinical study provides important additional data on the translatability of results from a non-clinical model to human subjects with regard to injection devices.

## MATERIALS AND METHODS

### Animals

Each of the two non-clinical studies that comprised this investigation used 18 female Yucatan miniature pigs (*Sus scrofa domestica*). All animals were over 4 months of age and were fed twice daily (a.m. and p.m.) except for study day (p.m. only). They were acclimated to the study room, which was set to maintain a temperature of ~ 17–27°C and a relative humidity of 40–70%, with a 12-h light 12-h dark cycle, for a minimum of 8 days before receiving SC injections. Animal health was routinely assessed by visible inspection, physical touch, monitoring of food consumption, overall activity, and body weight, which ranged from 20 to 24 kg at the time of the procedure.

All animal experiments were conducted in full compliance with local, national, ethical, and regulatory principles and local licensing regulations under approved Institutional Animal Care and Use Committee (IACUC) protocols following the United States Department of Agriculture (USDA) guidelines and regulations for research.

### Materials

In the non-clinical studies, two test solutions were administered to Yucatan miniature pigs anesthetized with isoflurane gas. The formulations for the two solutions comprised the same components, based on a citrate buffer (pH = 5.5) with sodium carboxymethylcellulose (NaCMC), except that one test solution contained rHuPH20 at 2000 U/mL. The NaCMC was used to increase viscosity, which was found to be 6 centipoise (cP) at 20°C for the final test solution (measured using Brookfield Cone/Plate Viscometer). Solutions of 1 or 2 mL were administered depending on the injection type (described in next section). In the clinical study, no active drug was administered; all subjects received 2 mL SC injections of a sterile citrate buffer matrix (pH = 6) that contained inactive excipients. The viscosity of the final test solution was 1 cP. The buffer matrix was manufactured and filled into the PFS using good manufacturing practices, providing a sterile product appropriate for clinical trial use. Composition of the buffer matrices used for the non-clinical and clinical studies are shown in Supplementary Table I.

### Auto-Injector Devices

Three spring-based auto-injector platforms of proprietary design were used in this investigation. The fully automated 2-mL auto-injector (AI2) delivers a 2-mL volume at an injection depth of 5.5 mm. It provides needle auto-insertion, auto-injection of the test article, and needle auto-retraction. The semi-automated 2-mL auto-injector device (sAI2) provides auto-injection of the test article but requires both manual needle insertion and retraction. It can be adjusted for needle insertion depths of 5.5 and 7.5 mm (sAI2/5.5 and sAI2/7.5). Finally, the fully automated 1-mL auto-injector (AI1) delivers a volume of 1 mL at an injection depth of 5.5 mm.

Both fully and semi-automated 2-mL auto-injectors (AI2 and sAI2 devices) were adapted to deliver a 2-mL volume in approximately 4–8 s for 15–30 mL/min or 15–25 s for 5–8 mL/min. The delivery speeds were adjusted by using various drive spring forces.

Pre-filled syringes with volumes of 1 mL (PFS1) or 2 mL (PFS2) were also compared with the auto-injection devices. All auto-injectors and pre-filled syringes utilized a 27G needle. Configurations of all devices used in the clinical and non-clinical studies are summarized in Supplementary Tables II and III.

### Non-clinical Study Design

This investigation comprises two non-clinical studies. In the first, 18 female Yucatan miniature pigs were injected with a citrate/NaCl buffer matrix combined with NaCMC in the presence and absence of rHuPH20 using the AI2 and sAI2 devices. In the second, 18 additional miniature pigs were injected with the citrate/NaCl buffer matrix combined with NaCMC in the presence and absence of rHuPH20 using the AI1 and 1- and 2-mL PFS devices. To evaluate each AI configuration, 2 cycles of dosing were used in the first non-clinical study with a 1-week recovery period in between (*n* = 6/group), and 1 cycle was used in the second study. Each animal received two SC injections of a specified test article into the abdominal region per dosing cycle. Injection sites were located on the left and right abdominal regions, approximately 3 cm toward the midline starting from the cranial end of the inguinal fold and then approximately 6 cm cranial. In both non-clinical studies, injection devices were randomly assigned to each animal. If animals received buffer alone (placebo), this was administered to one injection site, while the contralateral site received the buffer in combination with rHuPH20. The device with the test article without rHuPH20 was administered first to each animal.

Once the injection sites were marked with a permanent marker, pre-injection photographs were obtained using a Vectra^®^ H1 high resolution 3D camera (Canfield Scientific, Parsippany, NJ, USA). Pre- and postinjection ultrasound scans of the injection site were obtained by recording scans of the site in two orthogonal axes using a Vevo 3100 Ultrasound (VisualSonics, Toronto, ON, Canada). Ultrasound images were used to assess the postinjection fluid height by measuring the distance from the top to the bottom of the fluid pocket in the postinjection ultrasound images.

Back leakage for each injection was measured by collecting fluid using a pre-weighed Visitec^®^ eye spear (Becton-Dickinson, Franklin Lakes, NJ, USA). Fluid from back leakage was collected for an interval of 30 s immediately following injection. The eye spear was re-weighed on an analytical balance with a precision of 0.1 mg, and the difference from its original weight was calculated.

A Panasonic Lumix DMC-FZ1000 high-speed video camera with an acquisition rate of 120 frames/s was used to record and measure drug delivery injection time. The fluid delivery injection time was defined as the amount of time from the initiation of the injector until the fluid was completely delivered and did not include the time required for needle insertion or retraction.

Local injection site swelling (bleb and edema) area and volume were measured using a digital caliper when visible. After caliper measurements, photographs of the injection site were obtained using a standard digital camera (Canon PowerShot S120) and a 3D camera (Canfield Vectra^®^ H1). Finally, the injection site was qualitatively assessed postinjection for size of swelling, firmness, and erythema by three independent scorers using five-point scoring systems (Supplementary Table IV). The incidence of erythema, measured as a percentage proportion of injection sites showing erythema, was also recorded for the AI2 and sAI2 devices using the five-point scoring system.

### Calculation of Local Swelling Volume and Area

Volume and area of postinjection swelling were measured using both caliper measurement and 3D camera image analysis. Digital calipers were used to measure the maximum length, width, and height of the swelling forming postinjection. The shape of the swelling that lies above the skin is only the upper half of an ellipsoid; thus, the formula used to calculate swelling volume is half the volume of an ellipsoid: swelling volume = (2/3) × *π* × *A* × *B* × *C*, where *A *= length/2, *B* = width/2, and *C* = height.

The area of the swelling was calculated using the formula for the area of an ellipse: swelling area = π × *A* × *B,* where *A* = length/2 and *B* = width/2.

As an orthogonal approach for assessing the size of post infusion swelling, high definition pre- and postinjection 3D images of the injection site were obtained using a 3D camera. This provided an additional endpoint for assessing changes in volume, surface area, and height of the swelling over time that was not possible using caliper measurements. Validated software (38) associated with the camera was used to measure differences in height and volume between the pre- and postinjection surfaces. For injections administered using AI1, PFS1, and PFS2 devices, postinjection swelling resolution was also assessed by determining the height of the swelling using 3D images taken at 15, 30, 60, and 240 min postinjection.

### Clinical Evaluation of 2 mL SC Injections in Humans

#### Study Design

A single-center, open-label, clinical study was conducted to evaluate the AI2 and PFS2 devices for SC delivery in humans. The primary objective of the study was to compare the safety and tolerability of 2-mL SC injections into the abdomen of healthy male and female volunteers using the AI at fast and slow injection durations compared with the safety and tolerability of the PFS devices. Volunteers had to be willing to receive and/or self-administer injection into the skin. Key exclusion criteria included pregnancy/lactation, current use of aspirin or other nonsteroidal anti-inflammatory drugs, history or presence of a bleeding disorder, any condition that could affect pain perception from an injection, and excessive tattoos over the abdomen that would interfere with injection site assessments.

The study was a two-treatment arm, four-way crossover, partial replicate design comparing 2-mL volume SC injections into different abdominal quadrants. The treatment arms differed by administration of a second fast or slow injection (Supplementary Table V). Volunteers were evenly distributed between the two treatment arms and randomized within each treatment arm.

#### Study Drug Formulation and Administration

A sterile citrate/NaCl buffer matrix (pH = 6) was administered to 60 volunteers using the AI2 and PFS2 devices. rHuPH20 was not added to any of the administered solutions. The devices had the same specifications as those used in the Yucatan miniature pig investigation. The different speeds of 2-mL AIs were achieved *via* different drive springs (*i.e.*, fast AI used a higher drive spring force than slow AIs). The buffer solution had lower viscosity (1 cP) than the buffer used in the miniature pig investigation (6 cP). Volunteers self-administered test solutions using the AI, as trained by the investigative site staff, and received staff-administered PFS. Each volunteer received a total of four injections.

### Study Assessments

The 2-mL AI was evaluated at two injection durations; targeted injection duration was around 5 s for AI fast and 10 s for AI slow. Injection duration and delivery times were measured using a calibrated stopwatch. A video recorder was used to record the injection site and the device as it was being used. The starting time for the PFS injection was the time when the plunger rod was pressed down and was considered completed when all of the buffer matrix was injected. The starting time for the AI was when a “click” sound was heard after the injection button was pressed. The AI injection was completed when the second click sound was heard, which indicated that the needle had retracted from the volunteer’s skin.

Injection site reactions (ISRs) were proactively assessed using an ISR form for each volunteer at every timepoint. For the 0-, 15-, and 30-min time points, a study site staff member observed the injection site location for any ISR (bruising, bleeding, leakage [0 timepoint only], swelling, pruritus, and erythema). Injection site leakage was calculated as the difference between the mass of the filter paper before and after blotting the injection site. Injection site swelling severity was determined based on the height (mm) above normal skin and defined as mild (< 2 mm), moderate (2–5 mm), or severe (> 5 mm). Injection site erythema size was categorized as barely noticeable (less than 25 mm diameter), slight (25–50 mm diameter), moderate (51–100 mm diameter), or severe (> 100 mm diameter). Injection site erythema severity was categorized as noticeable but very mild redness, clearly red, bright red, dark with ulceration, or necrosis at the injection site. Injection site pruritus severity was categorized as mild (interferes occasionally with activity or occasionally delays falling asleep), moderate (interferes frequently with activity, delays falling asleep, and/or occasionally awakens from sleep), or severe (severely impairs activity, frequently delays falling asleep, and/or frequently awakens from sleep). Volunteers were asked to score their pain with each injection according to the 100-mm visual analog scale (VAS), with a score of 0 mm being no pain and a score of 100 mm being worst imaginable pain. Subjects were asked to rate pain immediately (time zero to within 5 min) following the start of the injection and at 15 and 30 min after injection.

All procedures involving human participants were approved by site-affiliated institutional review board in the USA, and all subjects provided written informed consent prior to any study procedures.

#### Statistical Analysis

Safety and tolerability parameters, including adverse events, vital signs, bruising, bleeding, injection site leakage, induration, swelling, pruritis, erythema, and injection duration, were listed and summarized using standard descriptive statistics. A statistical comparison of injection-site leakage between each of the injection methods (PFS, AI fast, and AI slow) was conducted using a linear mixed-effects model with a fixed effect for treatment, sequence, and injection number and using a random effect for volunteer within the sequence. The least squares mean for each injection, differences in least squares means between injections, and the 90% confidence interval for the difference were calculated. All tests were conducted at a two-sided alpha level of 0.1, unless otherwise stated. Data analyses were performed using JMP Version 12 (SAS Institute Inc., Cary, NC, USA).

## RESULTS

### AI Injection Time in Miniature Pigs

**Fig. 1:**
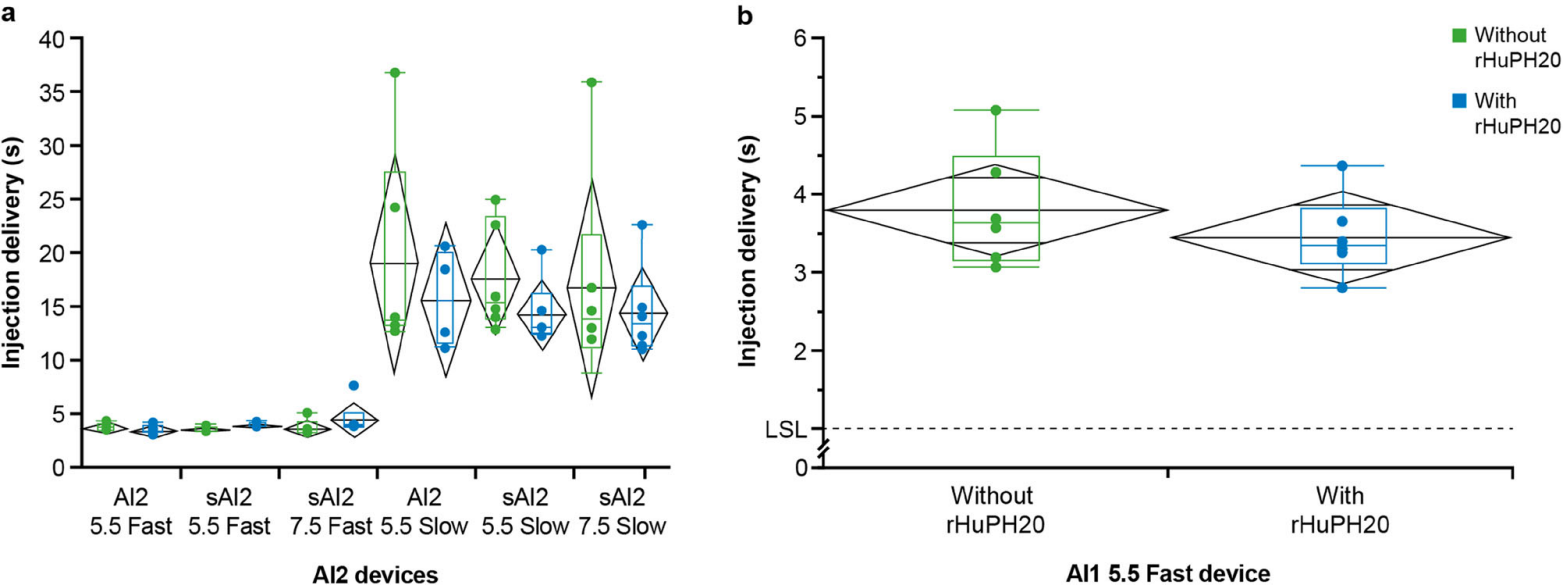
Injection delivery time in miniature pigs with and without rHuPH20. Delivery times with AI2 devices at fast and slow speeds (**a**) and AI1 device (**b**), with and without rHuPH20. *AI1* 1-mL auto-injector device, *AI2* 2-mL auto-injector device, *rHuPH20* recombinant human hyaluronidase PH20, *sAI2* semi-automated 2-mL auto-injector device

In miniature pigs, the addition of rHuPH20 resulted in reduced delivery times and variability for slow injections using the-2-mL AI or sAI devices (Fig. 1, Table I). The addition of rHuPH20 shortened the delivery time of 2-mL slow injections by 3–4 s (20–26% reduction; Fig. 1a, Table I) and shortened the delivery time of 1-mL AI injections by 1 s (22% reduction; Fig. 1b, Table I). The addition of rHuPH20 did not result in any difference in injection time for 1- and 2-mL AI fast injections. Overall, injection with the fast 2-mL auto-injection devices, with and without rHuPH20, had significantly reduced delivery times and less variability than injection with the slower 2-mL devices (Fig. 1a).

**Table I:** Injection Delivery Time and Variability in Miniature Pigs, With and Without rHuPH20

Device	Mean injection time without rHuPH20 (seconds [±SEM])	Mean injection time with rHuPH20 (seconds [±SEM])	Variability without rHuPH20 (%)	Variability with rHuPH20 (%)*
AI2 5.5 slow	19.3 (4.0)	15.9 (2.3)	21	14
sAI2/5.5 slow	17.8 (2.0)	14.5 (1.3)	11	9
sAI2/7.5 slow	17.0 (4.0)	14.6 (1.7)	24	12
AI1 5.5 fast	3.8 (0.3)	3.5 (0.2)	8	6

*AI1* 1-mL auto-injector device, *AI2* 2-mL auto-injector device, *rHuPH20* recombinant human hyaluronidase PH20, *sAI2* semi-automated 2-mL auto-injector device, *SEM* standard error of the mean

*Percent variability was calculated by dividing the SEM with the mean

### Swelling at the Injection Site in Miniature Pigs

**Fig. 2:**
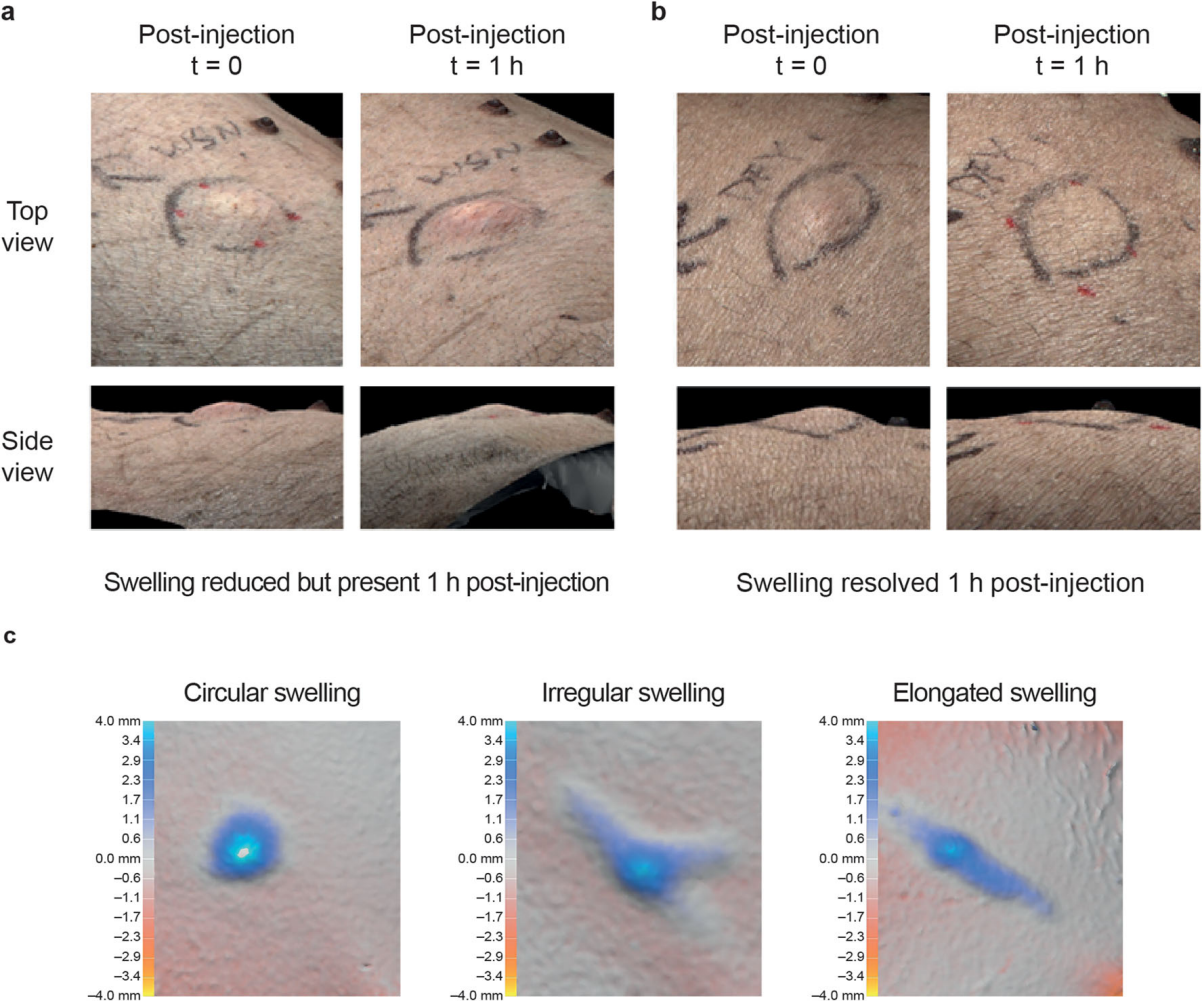
Representative images of swelling formation in Yucatan miniature pigs following injection with 2-mL sAI2 auto-injectors. Swelling may be present but slightly reduced 1 h after injection using sAI2/5.5 (**a**) or resolved 1 h after injection using sAI2/7.5 when co-administered with rHuPH20 (**b**) and of circular or elongated shape (**c**). *rHuPH20* recombinant human hyaluronidase PH20, *sAI2* semi-automated 2-mL auto-injector device

**Fig. 3:**
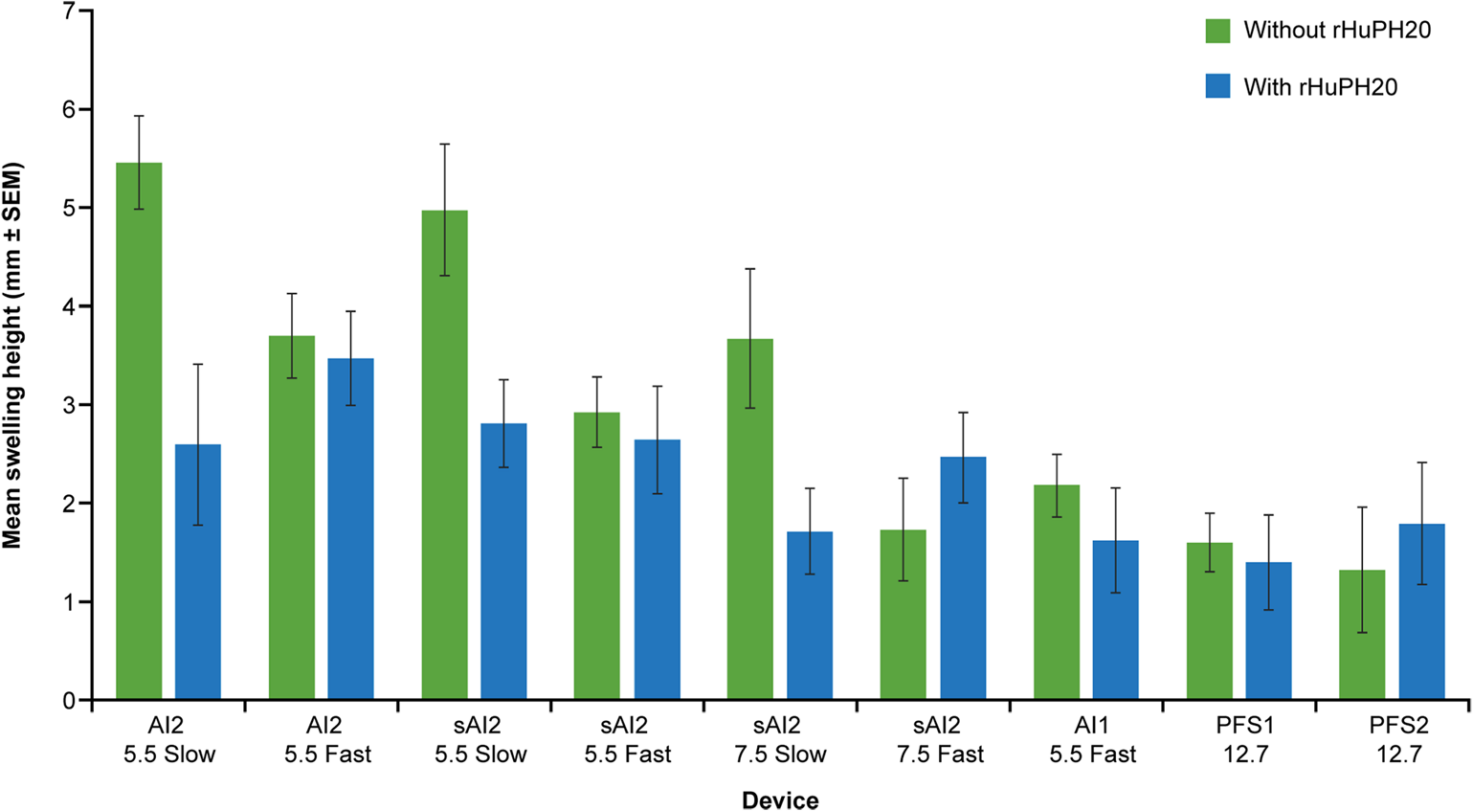
Mean postinjection swelling height with and without rHuPH20 using different devices in miniature pigs. After completion of each injection, the local injection site swelling was marked if visible and measured using a digital caliper. *AI1* 1-mL auto-injector device, *AI2* 2-mL auto-injector device, *PFS1* 1-mL pre-filled syringe, *PFS2* 2-mL pre-filled syringe, *rHuPH20* recombinant human hyaluronidase PH20, *sAI2* semi-automated 2-mL auto-injector device, *SEM* standard error of mean

Injection of 2 mL NaCMC solution of 6 cP viscosity using the AI2 and sAI2 devices resulted in noticeable moderate-to-severe swelling of a circular or elongated shape in miniature pigs (Fig. 2). Postinjection swelling heights for injections administered with the different devices, with and without rHuPH20, are reported in Fig. 3. Among the devices and administrations, swelling height was most pronounced without rHuPH20 with the slow injections for AI2 and the sAI2/5.5 (Fig. 3), where low drive spring forces are likely insufficient to push solution into the deep SC space, resulting in a more noticeable swelling. Injections facilitated by rHuPH20 mostly showed lower mean swelling heights in comparison to injections without rHuPH20, particularly all three slow injections of weak spring forces (AI2, sAI2/5.5, and sAI2/7.5). These differences were not seen with injections administered with sAI2/7.5 fast and PFS2 devices, where high enough forces, whether from the spring or hand, are involved to overcome tissue backpressure.

**Fig. 4:**
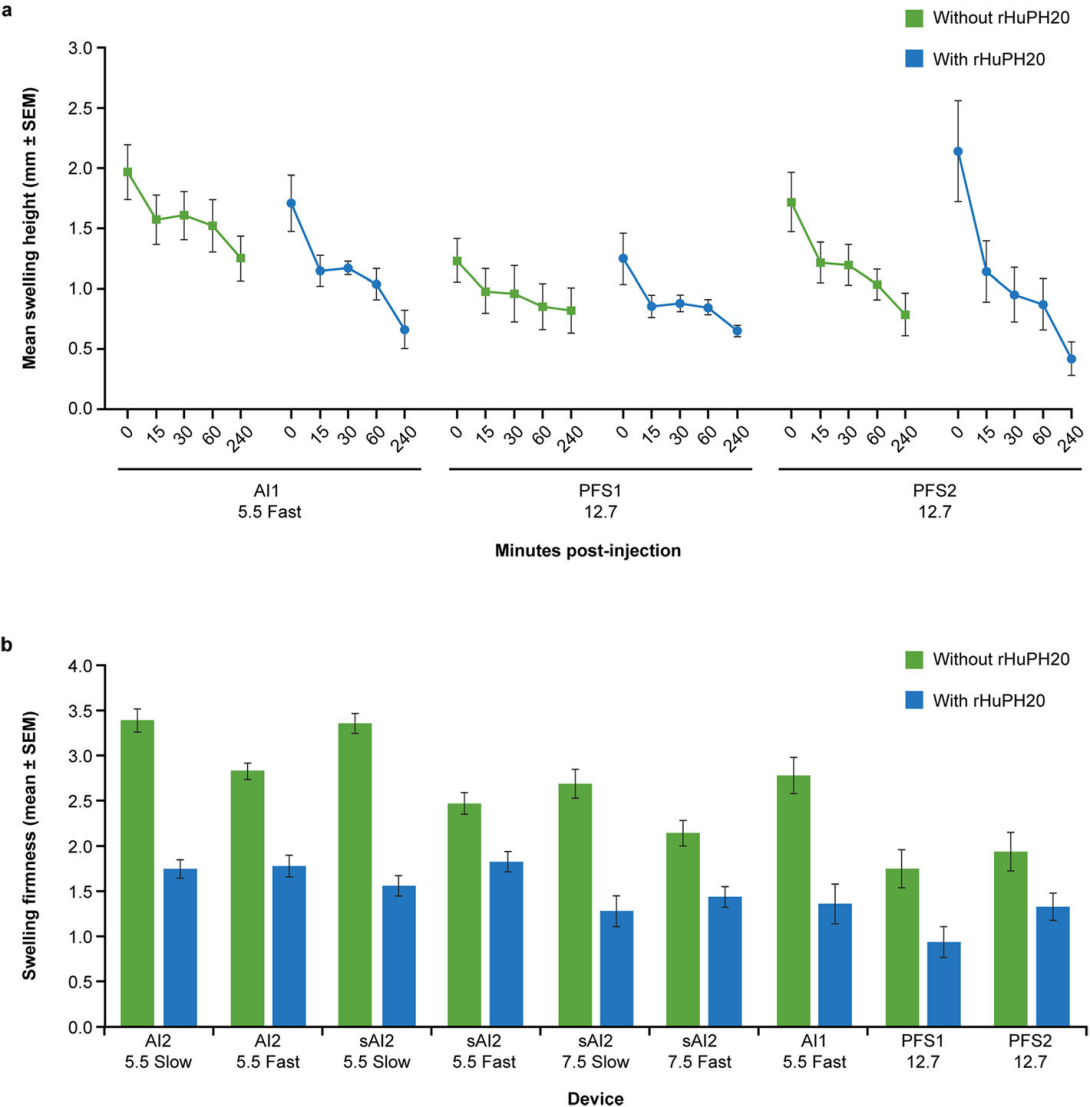
Mean postinjection swelling height and firmness in miniature pigs. Side-by-side comparison of actual swelling heights (mm ± SEM) over time for AI1, PFS1, and PFS2 devices (**a**). Postinjection swelling firmness with or without rHuPH20 for all devices (**b**). *AI1* 1-mL auto-injector device, *AI2* 2-mL auto-injector device, *PFS1* 1-mL pre-filled syringe, *PFS2* 2-mL pre-filled syringe, *rHuPH20* recombinant human hyaluronidase PH20, *sAI2* semi-automated 2-mL auto-injector device, *SEM* standard error of mean

Swelling resolution was assessed using 3D camera images taken at 15, 30, 60, and 240 min postinjection with AI1, PFS1, and PF2 devices, with and without the addition of rHuPH20. Comparison of mean swelling height over time demonstrated that the height of swelling resulting from injections of NaCMC solution containing rHuPH20 consistently declined faster than mean swelling volume resulting from devices that contained the NaCMC solution alone (Fig. 4a). Notably, while the immediate postinjection height was greatest for the PFS2 device with rHuPH20, this group also experienced the greatest total height decrease compared with all other device configurations.

Swelling firmness was assessed *via* manual palpation immediately postinjection with AI1, AI2, sAI2, PFS1 and PFS2, devices, with and without the addition of rHuPH20. Swelling firmness immediately postinjection was clearly reduced for injections with rHuPH20 *versus* without rHuPH20, particularly for the slow injections (Fig. 4b) and for injectors with shorter needle lengths.

### Erythema at the Injection Site in Miniature Pigs Following SC Injection

**Fig. 5:**
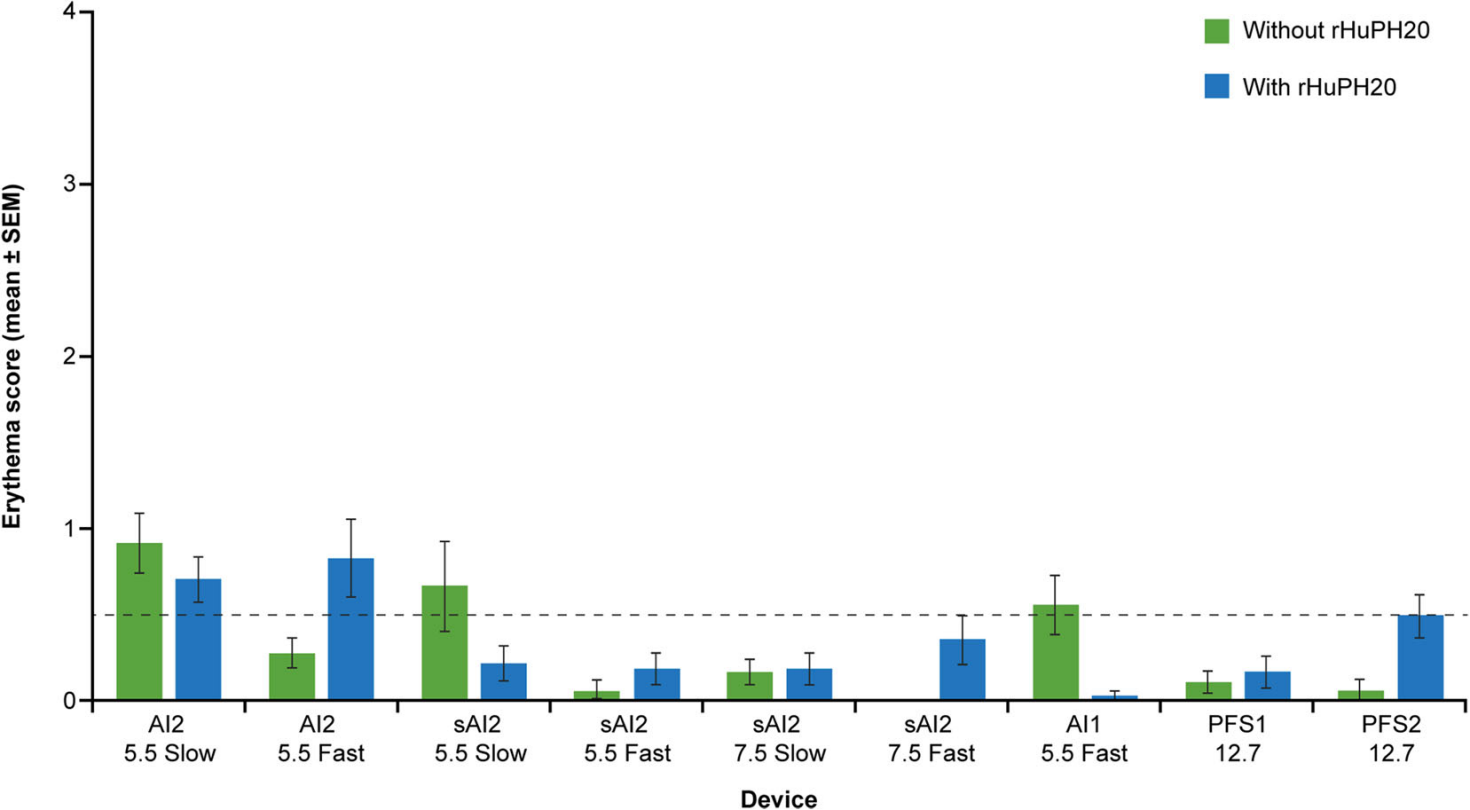
Mean erythema score of swelling in miniature pigs following injection with different devices with and without rHuPH20. *AI1* 1-mL auto-injector device, *AI2* 2-mL auto-injector device, *PFS1* 1-mL pre-filled syringe, *PFS2* 2-mL pre-filled syringe, *rHuPH20* recombinant human hyaluronidase PH20, *sAI2* semi-automated 2-mL auto-injector device, *SEM* standard error of mean

The proportion of injection sites showing erythema in miniature pigs and the mean erythema score, assessed by three different scorers using a five-point scoring system, for the different injection devices are shown in Fig. 5. Mean erythema score was low for all devices (< 1.0), indicating very slight, barely noticeable erythema. *Post hoc* analysis of the erythema severity data showed that all instances of erythema in miniature pigs corresponded with the “slight” category used for classifying the human erythema data. The higher erythema score injections (*e.g.*, > 0.5) are primarily related to shallow injections (5.5 mm).

### Back Leakage from SC Injections in Miniature Pigs

**Fig. 6:**
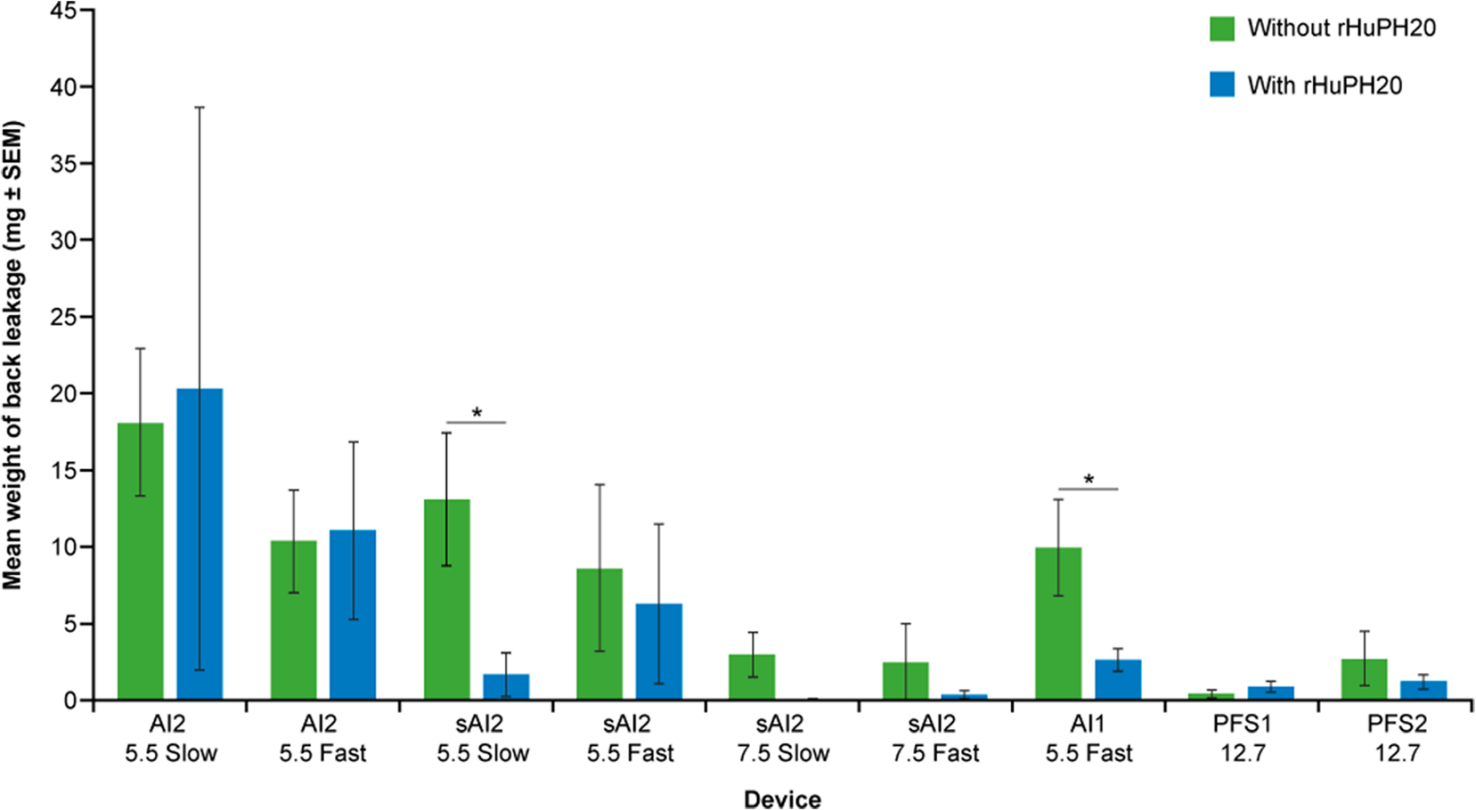
Back leakage from SC injections in miniature pigs by injection device. Immediately postinjection, any back leakage from the injection site was collected for an interval of 30 s using an eye spear, which was then immediately weighed, and the weight recorded. *P < 0.05. *AI1* 1-mL auto-injector device, *AI2* 2-mL auto-injector device, *PFS1* 1-mL pre-filled syringe, *PFS2* 2-mL pre-filled syringe, *rHuPH20* recombinant human hyaluronidase PH20, *sAI2* semi-automated 2-mL auto-injector device, *SC* subcutaneous, *SEM* standard error of mean

Back leakage for injections with different devices is shown in Fig. 6. Minor back leakage (< 1% of total delivery volume) was observed for most of the 2-mL miniature pig injections. With AI delivery, deeper injections (7.5 mm) resulted in a lower amount of back leakage compared with shallower injections (5.5 mm). The level of back leakage with deeper AI injections was similar to the leakage with PFS injections. Lower back leakage resulting from the addition of rHuPH20 was observed for some of the device configurations but not others.

### Clinical Tolerability Evaluation for 2-mL SC Injections in Humans

A total of 60 healthy volunteers participated in the clinical tolerability evaluation of 2-mL SC injections of a sterile buffer (without rHuPH20) and were randomly assigned to treatment. Mean (standard deviation [SD]) age was 44 (13) years (range 18–71 years). Sixty-eight percent of the volunteers were female. Mean (SD) body mass index was 27.8 (4.7) kg/m^2^. All volunteers received at least one injection; one volunteer did not complete the study due to an adverse event of presyncope deemed unrelated to treatment.

**Fig. 7:**
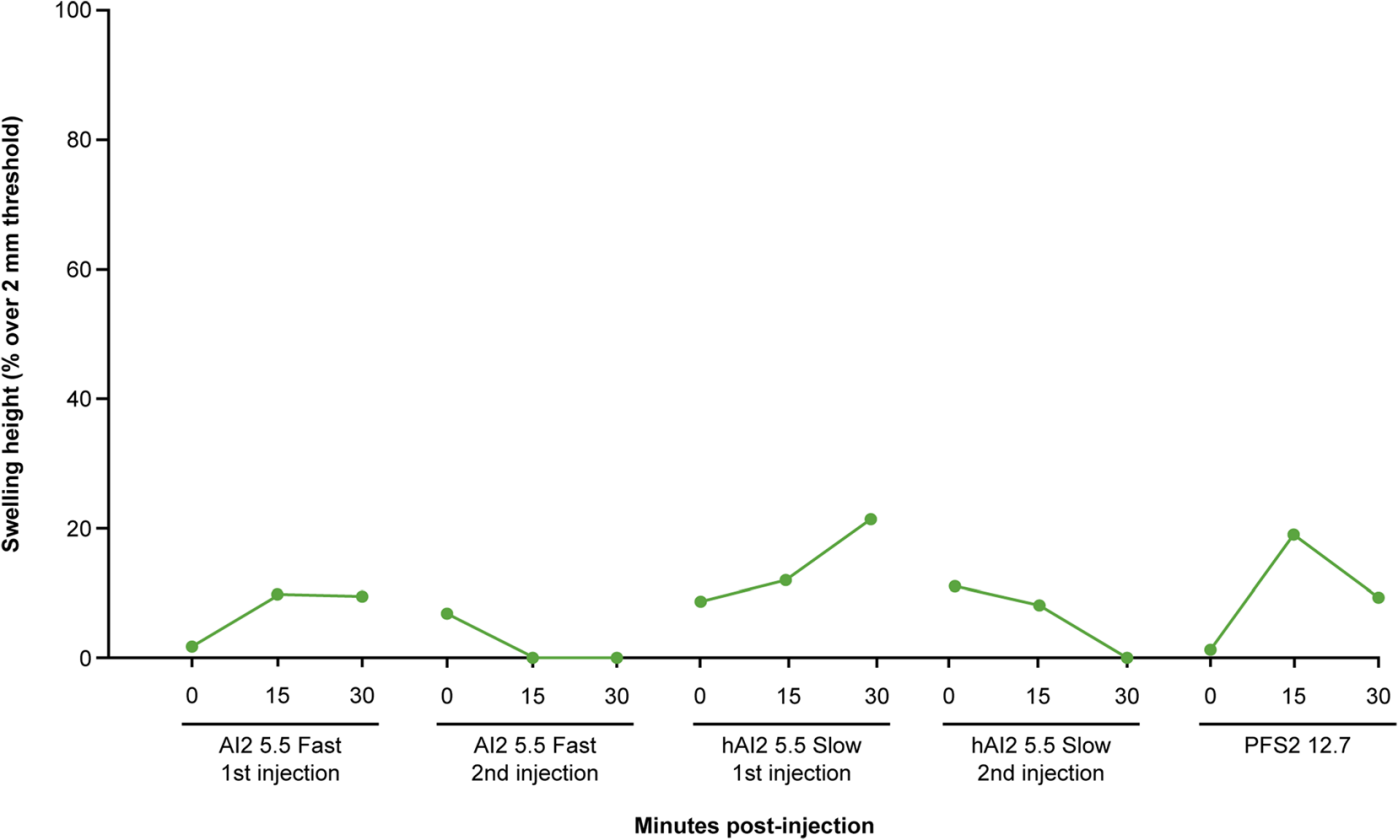
Injection site swelling following injection with AI2 or PFS2 devices in human volunteers. Swelling height was measured using digital calipers. Moderate and severe swellings are depicted as a percentage over a 2-mm threshold. *AI2* 2-mL auto-injector device, *hHI2* human 2-mL auto-injector device, *PFS2* 2-mL pre-filled syringe

In human volunteers, non-mild swelling was measured as a percentage over a 2-mm height threshold, which was considered moderate at 2–5 mm or severe at > 5 mm. In general, low injection-site swelling (< 22% moderate or severe) was observed following injection with different devices (Fig. 7). The most prominent swelling immediately after injection was observed with AI2 slow injection, followed by AI2 fast and then PFS2 injection. This trend is consistent with results in miniature pigs.

The back leakage observed for 2-mL injections in humans was negligible (Supplementary Fig. 1) and much lower than observed in the miniature pig studies. The mean leakage values were very small or negative (potentially due to liquid evaporation), suggesting that the fluid mass was within the range of measurement variability. The low fluid leakage results from our 2-mL clinical injections are consistent with a recent report of 2-mL SC injections in humans (39).

**Fig. 8:**
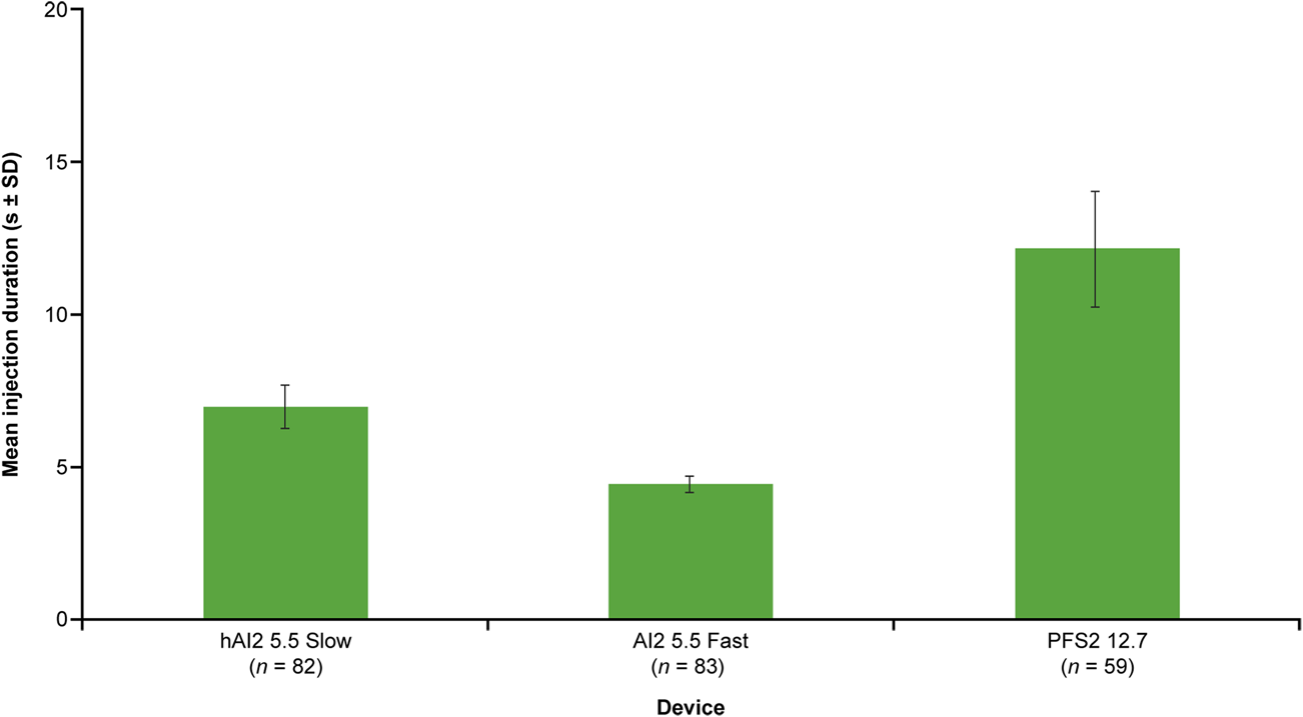
Injection duration for 2-mL devices used in the clinical trial. *AI2* 2-mL auto-injector device, *hHI2* human 2-mL auto-injector device, *PFS2* 2-mL pre-filled syringe, *SD* standard deviation

In terms of injection duration (Fig. 8), the human AI2 (hAI2) slow device had a mean injection time of 7 s with a moderate variability (SD, 0.70 s) compared with AI2 fast, which had a shorter, 4-s injection time with a tighter distribution (SD, 0.25 s). PFS2 has the longest injection time (mean = 12 s) and, as expected, the largest variability (1.88 s), due to the lack of mechanical control for the injection speed.

**Fig. 9:**
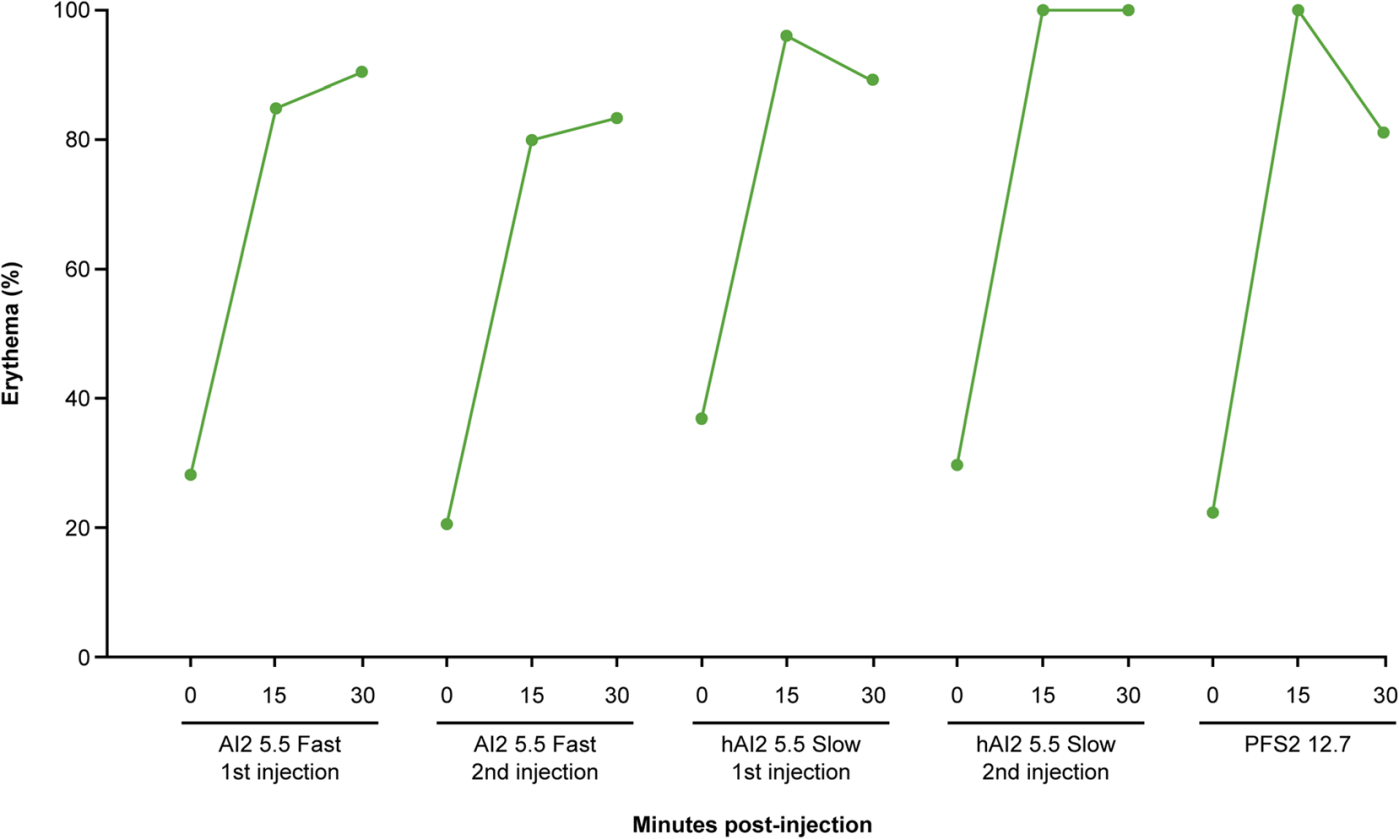
Percentage incidence of erythema at 0, 15, and 30 min following injection with 2-mL devices in human volunteers. *AI2* 2-mL auto-injector device, *hHI2* human 2-mL auto-injector device, *PFS2* 2-mL pre-filled syringe

In humans, 22–40% of volunteers showed erythema immediately following injections (Fig. 9). Incidence of erythema increased to approximately 80–100% of volunteers within 15 min following injection and persisted through to the 30-min time point. Note these erythema findings are from solicited, prospective observation of the injection sites, which is expected to result in higher rates than the typical levels of spontaneous, self-reported injection site reactions including erythema in most clinical trials. The majority of incidences of erythema (60–84%) were classed as mild. There were few moderately severe cases (clearly red) and no severe cases (bright red or dark with ulceration or necrosis at the injection site).

The frequency of bruising was generally low across injection methods, occurring in 0–8.3% of volunteers at 30 min postinjection. While immediate bruising only occurred with PFS injections, bruising was noted in a greater number of volunteers at follow-up than on the study day. The majority of volunteers had no injection site pruritus with any of the injection methods, and there were no obvious trends with regard to injection method among subjects who had injection site pruritus. All cases of injection site pruritus were mild. Injection pain intensity measurement, based on VAS, shows the presence of substantial moderate (30–70 mm VAS) or severe pain (70–100 mm VAS) irrespective of device type (PFS or AI) or injection speed (fast or slow). This is consistent with literature reports of citrate formulation injections (40), as the acute, irritant-induced injection pain is primarily driven by formulation composition.

## DISCUSSION

Data from a miniature pig model were used to evaluate different injection devices for SC delivery of a viscous fluid (6 cP placebo citrate buffer with sodium NaCMC) with and without the addition of rHuPH20. Additional data investigating several of similar devices for SC delivery of fluid (1 cP citrate buffer matrix without rHuPH20) in humans enabled evaluation of the translatability of the non-clinical miniature pig as a model for human clinical results.

Overall, tolerability of injections into the miniature pig abdomen with 6 cP placebo citrate buffer matrix was found to be adequate at the tested ranges of injection speed and depth for all devices. In the miniature pig model, swelling was moderate-to-severe in most cases but softened and resolved within a few hours. Overall, the swelling height was lower with fast AI injection compared with slow AI injection. This difference is likely due to the high drive spring force associated with fast AI injections, driving the injected solution deeper into the SC space than with slow AI injections and resulting in smaller swelling. In comparison, 2-mL PFS showed less swelling than the 2-mL AIs in general, possibly due to deeper SC injections with longer exposed needle (12.7 mm × Sin [45^0^] = 10.8 mm for PFS, *vs.* 5.5 mm perpendicular to skin for AI) and more persistent pressure forcing the liquid into the SC space. The AI1 and 1-mL PFS devices all showed similar modest postinjection swelling, which was less than that observed for most of the 2-mL injections in the miniature pig model. This finding is not unexpected, given the 50% reduction in dosing volume. Deeper injections (7.5 mm) resulted in a slightly lower swelling height than the shallower injections (5.5 mm) delivered at the same speed, possibly due to more downward liquid flow toward the deeper SC space.

Addition of rHuPH20 resulted in slightly faster injection times and reduced variability for the slower speed sAI2 and AI2 devices that employed a low spring force. This reduction in injection time was likely due to rHuPH20 degrading the HA in the extracellular matrix of the SC space, permitting increased bulk fluid flow (20,21). However, addition of rHuPH20 did not significantly reduce the amount of time required for the administration of 1 or 2 mL for the faster injections that utilized a higher spring force. The tissue backpressure that is caused, in part, by the presence of HA is overcome by these high spring forces. Degradation of HA by rHuPH20, therefore, makes little difference when high spring forces are utilized for the injection of the volumes tested in this study. rHuPH20-facilitated injections also showed reduced swelling size in comparison to injections without rHuPH20 for most devices and among those devices tested (AI1, PFS1, and PFS2), the addition of rHuPH20 resulted in more rapid swelling resolution and reduction in swelling firmness. It is important to note that the difference in AI injection time for slow injections with or without rHuPH20 indicates that there is an almost instantaneous onset of enzymatic activity for rHuPH20. This temporal enzymatic effect for rHuPH20 upon SC injection and the more rapid swelling resolution have not previously been published. These results suggest that rHuPH20 may improve SC injections delivered at the fast injection rates involved (10–30 mL/min). This quick enzymatic action suggests an intriguing possibility of using rHuPH20 in fast SC delivery of a large bolus of fluid (*e.g.*, 3–10 mL), to reduce the injection time for a wearable on-body injector from minutes or dozens of minutes to less than 1 min or even seconds.

Fluid back leakage was minimal and acceptable for all injections in miniature pigs and is < 0.9 and < 1.0% of the total delivery volume for 2- and 1-mL injections, respectively. Only very slight erythema was observed for all injections. The higher erythema score injections were primarily related to shallow injections (5.5 mm), possibly because local hyperemia in superficial capillaries is more visible with shallower tissue injury. In humans, SC injection of 1 cP citrate buffer matrix without rHuPH20 using AI2 or PFS2 devices resulted in insignificant leakage, low swelling, and mild erythema. Consistent with the miniature pig results, greater swelling was observed with slower-delivery AI2 devices that utilize lower spring forces than in faster-delivery AI2 devices.

Comparison of clinical data from humans and non-clinical data from miniature pigs allows some evaluation of the suitability of the miniature pig as a model for testing different SC injection devices such as AIs and PFS. While miniature pig and human injection site responses to different delivery schemes share a number of similarities, as demonstrated in these studies, there are some differences in injection site responses, which have been better characterized in our studies. First, swelling at the injection site is generally greater in miniature pigs than in humans (Figs. 3 and 7), owing to miniature pigs (typical weight 20–24 kg) having less SC space than humans (> 50 kg). Second, miniature pigs show greater, although still minor, back leakage of injected liquid than the negligible amount seen in the human volunteers (Fig. 6). This higher leakage in miniature pigs, related to increased swelling, suggests higher tissue backpressure at the abdomen injection sites in miniature pigs than in humans, which is in part due to differences in body weight and relative age between the human volunteers and the miniature pigs. While juvenile or adolescent pigs were used in this study, the age range of the humans who received SC injections was much broader. As skin aging is associated with physiological changes, including reduced elasticity and turgidity (41–43), the relatively more elderly skin of the human volunteers was likely looser, potentially reducing tissue backpressure, which in turn could allow greater absorption and reduced leakage. Finally, the overall severity of erythema was lower in miniature pigs, in which no incidence of erythema was classed higher than “slight,” whereas in humans, some incidences of erythema were classed as “moderate” severity. This is likely due to the reduced hyperemia of superficial capillaries in response to local injury (needle penetration) in anesthetized pigs. In addition, the placebo in the clinical study contained L-arginine (Supplementary Table I), which is known to induce nitric oxide-dependent vasodilation in healthy human patients (44). This may explain the greater severity of erythema in humans. Furthermore, erythema was scored by different investigators in the clinical and non-clinical studies, which may contribute to the disparity in erythema severity between miniature pigs and humans. In summary, non-clinical data from miniature pigs may provide helpful directional guidance for extrapolating to humans. For some endpoints, such as swelling and back leakage, the miniature pig exaggerates the reaction observed in humans and could act as a more rigorous model for worst-case testing.

Limitations of this investigation include the lack of direct comparison between miniature pigs and humans; that is, the studies were carried out separately, thus making comparisons between the species inappropriate for some endpoints. Importantly, the human volunteers were only administered placebo doses, without the rHuPH20 component. Furthermore, the injected placebo in the human study had much lower viscosity (1 cP) than that used in the miniature pigs (6 cP) and contained L-arginine, a known vasodilator (44), which may induce erythema when injected subcutaneously. In addition, the two 2-mL AI slow configurations are different in drive spring forces (44% reduction in pig studies), so the injection durations are very different (6–8 s in humans *vs.* 10–35 s in pigs). Lastly, injection site assessment methodology, criteria, and operation staff training are different in tolerability assessments between pig and human studies. All these differences should be considered when interpreting the results. However, our studies clearly show consistent injector performance between humans and miniature pigs when using the same device configuration.

## CONCLUSIONS

The tolerability of the 1- and 2-mL auto-injection and PFS devices was found to be acceptable in miniature pigs, with moderate swelling, insignificant leakage, and slight erythema for most injections. The studies demonstrated that fast AI injection reduced swelling height compared with slow AI injection. Deeper injections were also found to produce a slightly lower swelling height than the shallower injections delivered at the same speed. The addition of rHuPH20 resulted in an improved injection, with lower swelling with most injection devices and reduced injection times for devices utilizing a lower spring force, suggesting an almost instantaneous onset of action for rHuPH20 enzymatic reactions. In addition to characterizing the performance of different SC injection devices, our findings suggest that the Yucatan miniature pig may be a useful non-clinical model for SC injection tolerability assessment and indicate that this species shows potential for clinical translation to humans for analysis of different injection device configurations.

## Supplementary Information

ESM 1 (DOCX 73.5 kb)
