# Supplementary material for: Subcutaneous Injection Performance in Yucatan Miniature Pigs with and without Human Hyaluronidase and Auto-injector Tolerability in Humans
Source: AAPS PharmSciTech. 2021 Jan 6;22(1):39. doi: 10.1208/s12249-020-01880-0 (PMC7788039; doi:10.1208/s12249-020-01880-0)
Supplement: Supplementary file 1 — (DOCX 73.5 kb) [file 12249_2020_1880_MOESM1_ESM.docx]

# Supplementary Materials

Supplementary Table I. Buffer Matrix Ingredients

|  | **Clinical study** | **Non-clinical studies** | |
| --- | --- | --- | --- |
| **Component** | **Citrate saline buffer (mg/mL)** | **Citrate buffer without rHuPH20 (mg/mL)** | **Citrate buffer with rHuPH20 (mg/mL)** |
| Sodium chloride | 5.8 | 8.77 | 8.77 |
| Polysorbate 80 | 0.4 | 0.3 | 0.3 |
| L-Arginine HCl | 10.5 | – | – |
| Sodium citrate dihydrate | 2.66 | 2.35 | 2.35 |
| Citric acid anhydrous | 0.17 | 0.385 | 0.385 |
| NaCMC | – | 10 | 10 |
| rHuPH20 | – | – | 2000 U/mL |
| Methionine | – | 1.49 | 1.49 |

HCl, hydrogen chloride; NaCMC, sodium carboxymethylcellulose; rHuPH20, recombinant human hyaluronidase PH20.

Supplementary Table II. Device Configurations in Miniature Pig Studies

| **Device abbreviation** | **Description** | **Volume delivered (mL)** | **Injection depth (mm)** | **Needle bevel** | **Target injection duration (s)*** |
| --- | --- | --- | --- | --- | --- |
| AI2 | Fully automated autoinjector | 2 | 5.5 | 27G | Slow (15 s)  Fast (5 s) |
| sAI2/5.5 | Semi-automated autoinjector | 2 | 5.5 | 27G | Slow (15 s)  Fast (5 s) |
| sAI2/7.5 | Semi-automated autoinjector | 2 | 7.5 | 27G | Slow (15 s)  Fast (5 s) |
| AI1 | Fully automated autoinjector | 1 | 5.5 | 27G | 4−5 |
| PFS1 | Pre-filled syringe | 1 | 12.7^†^ | 27G | 6 |
| PFS2 | Pre-filled syringe | 2 | 12.7^†^ | 27G | 10 |

AI1, 1-mL auto-injector device; AI2, 2-mL auto-injector device; PFS1, 1-mL pre-filled syringe, PFS2, 2-mL pre‑filled syringe; sAI2, semi-automated 2-mL auto-injector device.

*Original target injection durations, which are different from real performance data.

^†^PFS injection depth is not as precisely controlled as with AIs, as it involves a 45⁰ angle penetration with a 12.7‑mm exposed length needle through pinched up skin, unlike vertical penetration of the AI needle without skin pinching.

Supplementary Table III. Device Configurations in Clinical Study

| **Device abbreviation** | **Description** | **Volume delivered (mL)** | **Injection depth (mm)** | **Needle bevel** | **Target injection duration (s)*** |
| --- | --- | --- | --- | --- | --- |
| AI2 fast | Fully automated autoinjector | 2 | 5.5 | 27G | 5 |
| hAI2 slow^†^ | Fully automated autoinjector | 2 | 5.5 | 27G | 10 |
| PFS2 | Pre-filled syringe | 2 | 12.7^‡^ | 27G | 10 |

AI2, 2-mL auto-injector device; hAI2, human 2-mL auto-injector device; PFS2, 2-mL pre-filled syringe.

*Original target injection durations, which are different from real performance data.

^†^hAI2 slow use a stronger drive spring than the AI2 slow used in the miniature pig studies, hence a shorter injection duration (7 s target instead of 15 s).

^‡^PFS injection depth is not as precisely controlled as within AIs, as it involves a 45⁰ angle penetration with a 12.7‑mm exposed length needle through pinched up skin, unlike vertical penetration of the AI needle without skin pinching up.

Supplementary Table IV. Grading Scales for Non-clinical Miniature Pig Studies for Erythema (a), Swelling Size (b), and Firmness (c)

**a)**

| **Scale** | **Description** |
| --- | --- |
| 0 | No erythema |
| 1 | Very slight erythema (barely perceptible) |
| 2 | Well-defined erythema |
| 3 | Moderate-to-severe erythema |
| 4 | Severe erythema (beet redness) to slight eschar formation |

**b)**

| **Scale** | **Description** |
| --- | --- |
| 0 | No swelling |
| 1 | Very slight swelling |
| 2 | Slight swelling |
| 3 | Moderate swelling |
| 4 | Severe swelling |

**c)**

| **Scale** | **Description** |
| --- | --- |
| 0 | No perceptible difference in firmness after injection |
| 1 | Very slightly firm (barely perceptible) |
| 2 | Mildly firm |
| 3 | Moderately firm |
| 4 | Very firm |

Supplementary Table V. Study Design Treatments for Clinical Evaluation of
2-mL Subcutaneous Injections in Humans

| **Treatment arm** | **Injections into the abdomen (sequence randomized)** |
| --- | --- |
| 1 | 1 x PFS2  2 x AI2 fast  1 x hAI2 slow |
| 2 | 1 x PFS2  1 x AI2 fast  2 x hAI2 slow |

AI2, 2-mL auto-injector device; hAI2, human 2-mL auto-injector device; PFS2, 2-mL pre-filled syringe.

Duration between the injections was a minimum of 60 min.

Supplementary Fig. 1. Back leakage from 2-mL SC injections in humans by injection device


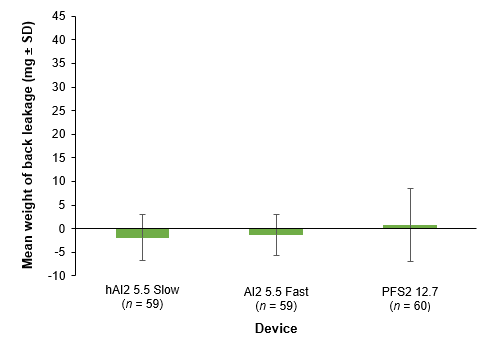


AI2, 2-mL auto-injector device; hAI2, human 2-mL auto-injector device; PFS2, 2-mL pre-filled syringe;
SC, subcutaneous; SD, standard deviation.

Negative values are potentially due to liquid evaporation and are within measurement variability.
